# Predicting mortality in non-traumatic intracerebral hemorrhage with glucose and lipid data

**DOI:** 10.3389/fendo.2026.1781456

**Published:** 2026-04-29

**Authors:** Qile Ye, Tongtong Xue, Yu Zhang, Ying Xu, Yuxin He, Jiayu Song, Xiangqi Meng, Ming Ye

**Affiliations:** 1 Department of Intensive Care Unit, The 2nd Affiliated Hospital of Harbin Medical University, Harbin, China; 2 Medical Examination Center, The 2nd Affiliated Hospital of Harbin Medical University, Harbin, China; 3 Department of Cranial Oncology Radiotherapy, Harbin Medical University Cancer Hospital, Harbin, China; 4 Department of Neurosurgery, The 2nd Affiliated Hospital of Harbin Medical University, Harbin, China

**Keywords:** blood glucose indicators, in-hospital mortality, LightGBM, lipid profile, non-traumatic intracerebral hemorrhage, XGBoost

## Abstract

**Aim:**

This study integrated dynamic glucose variation indicators and lipid profiles to develop and validate a machine learning-based predictive model for in-hospital mortality in patients with non-traumatic intracerebral hemorrhage (NTICH).

**Methods:**

Data of this study were derived from the Medical Information Mart for Intensive Care-IV (MIMIC –IV) database (2008–2019, which was split into training and internal validation sets at a 7:3 ratio) and from NTICH cases from the Second Affiliated Hospital of Harbin Medical University (for external validation). The Boruta algorithm was used to evaluate feature importance. Nine machine learning algorithms were used to develop predictive models for in-hospital mortality in NTICH patients. Model discrimination was assessed using the area under the curve (AUC) and clinical utility was evaluated with decision curve analysis. The SHapley Additive exPlanations (SHAP) method was used to rank feature importance.

**Results:**

A total of 2,323 patients were included from the MIMIC-IV database, with an in-hospital mortality rate of 14.03%. The Boruta algorithm identified 20 relevant features. The K-Nearest Neighbors model achieved the highest AUC in the training set (AUC = 0.980), the Light Gradient Boosting Machine (LightGBM) model performed best in the internal validation set (AUC = 0.851), and the eXtreme Gradient Boosting (XGBoost) model yielded the highest performance in the external validation set (AUC = 0.814). SHAP analysis identified that the Sequential Organ Failure Assessment score, Glasgow Coma Scale score, age, invasive mechanical ventilation, and mean glucose were the most important predictors.

**Conclusion:**

The XGBoost and LightGBM models demonstrated excellent performance for predicting in-hospital mortality in patients with NTICH. This study highlights the critical value of integrating dynamic glucose variation indices and comprehensive lipid profiles in improving the prognostic prediction of patients with NTICH. The identified key predictive factors provide actionable targets for early risk stratification and individualized intervention strategies, such as precise glucose regulation, thereby facilitating the optimization of resource allocation in neurocritical care and improving clinical outcomes.

## 1 Background

Non-traumatic intracerebral hemorrhage (NTICH) refers to bleeding resulting from the rupture of blood vessels within the cerebral parenchyma due to non-traumatic factors, which may extend into the ventricular system. As one of the most devastating subtypes of stroke, it accounts for 10%-15% of all stroke cases, with a higher prevalence of approximately 18%-20% among the Asian population (1). NTICH is characterized by high mortality and disability rates: the 30-day mortality rate can reach 44%-50%, the 1-year mortality rate exceeds 50%, and only 38% of survivors live beyond 1 year. Most survivors experience severe neurological deficits, placing a heavy burden on families and society (2, 3). Currently, commonly used clinical prognostic assessment tools, such as Glasgow Coma Scale (GCS) Score, mostly rely on traditional indicators such as hematoma volume and consciousness status. These tools fail to fully capture the dynamic pathophysiological changes that occur during disease progression and thus have limited predictive accuracy (4). Therefore, exploring more sensitive and dynamic biomarkers, as well as developing efficient predictive models, is crucial for optimizing the stratified management of NTICH patients and improving their prognosis.

Recent studies have shown that impaired glucose homeostasis and abnormal lipid metabolism play key roles in the prognosis of NTICH (5). Stress-induced hyperglycemia and blood glucose fluctuations can exacerbate secondary brain tissue injury by disrupting the blood-brain barrier, promoting inflammatory responses, and inducing oxidative stress (6). Traditional blood glucose testing only reflects instantaneous blood glucose levels, yet blood glucose is a rapidly changing parameter (7). In contrast, dynamic glucose variation index can capture dynamic indicators such as mean blood glucose, blood glucose standard deviation, and blood glucose difference between maximum and minimum values, thereby more comprehensively reflecting the body’s glucose metabolism status. Its predictive value has been verified in the field of ischemic stroke (8). The lipid profile, such as low-density lipoprotein cholesterol (LDL-C), high-density lipoprotein cholesterol (HDL-C), is not only a core risk factor for atherosclerotic cerebrovascular disease but also may be involved in prognosis regulation by affecting coagulation function, vascular repair, and inflammatory responses (9, 10). However, existing studies have mostly focused on individual lipid indicators, lacking a systematic analysis of the overall characteristics of the lipid profile.

With the rapid advancement of artificial intelligence technology, machine learning (ML) has demonstrated significant advantages in medical prognostic prediction (11). Studies based on large public databases such as Medical Information Mart for Intensive Care (MIMIC) have confirmed that ML algorithms can effectively integrate multi-dimensional clinical data and significantly improve the predictive accuracy of endpoints such as mortality in intracerebral hemorrhage patients (12). Nevertheless, existing models mostly rely on single-point laboratory tests at the early after admission and fail to fully leverage data obtained from multiple time-point measurements, particularly the combined features of dynamic glucose variation indices and lipid profiles. Therefore, this study intends to combine data from the MIMIC-IV database and the hospital center of our institution, innovatively integrate dynamic glucose variation indices and comprehensive lipid profile characteristics, to construct an ML-based predictive model for in-hospital mortality in NTICH patients. The aim is to provide evidence-based support for the early clinical identification of high-risk patients and the development of individualized intervention strategies (precise glucose regulation, lipid management), ultimately improving the quality of life of NTICH patients.

## 2 Methods

### 2.1 Data sources and ethics

This study primarily utilized data from the MIMIC-IV database and the Department of Critical Care Medicine, the Second Affiliated Hospital of Harbin Medical University. The MIMIC-IV database collects clinical data of more than 190,000 patients admitted to Beth Israel Deaconess Medical Center from 2008 to 2019, with 450,000 hospitalizations recorded. The database contains detailed information on patient demographics, laboratory tests, medications, vital signs, surgical procedures, disease diagnoses, medication management, and follow-up survival status. As MIMIC-IV is a public database, this study qualified for exemption from informed consent, and no additional informed consent from participants was required. To access the MIMIC-IV database, the authors completed the “Protection of Human Research Participants” course and obtained certification (Researcher Certificate Number: 73631027).

Data from the MIMIC-IV database were randomly split into a training set and an internal validation set at a ratio of 7:3. Medical records of ntICH patients admitted to the Department of Critical Care Medicine, the Second Affiliated Hospital of Harbin Medical University, between January 1, 2020, and September 1, 2025 were retrospectively analyzed for external validation of the predictive model. This study protocol was approved by the Institutional Review Board of the Second Affiliated Hospital of Harbin Medical University (Approval No: KY2025-371), and no informed consent from patients was required.

### 2.2 Inclusion and exclusion criteria

Inclusion Criteria:

1. Diagnosis of NTICH based on the International Classification of Diseases (ICD) coding system (9th or 10th revision).
2. Admission to the intensive care unit (ICU) during hospitalization.
3. Hospitalization duration > 72 hours.
4. Age ≥ 18 years.

Exclusion Criteria:

1. Insufficient blood glucose data (fewer than 3 measurements during ICU stay).
2. Patients with brain hemorrhage caused by various traumatic factors.
3. Missing values of key variables.

For patients admitted to the ICU multiple times, only data from the first ICU admission were included in this study.

### 2.3 Data collection

The primary outcome variable of this study was in-hospital mortality. The following indicators were collected: age, sex (female, male), marital status (divorced, married, single, widowed), race (black, other, white), height, weight; smoker (no, yes), alcohol abuse (no, yes), hypertension (no, yes), diabetes (no, yes), myocardial infarction (no, yes), congestive heart failure (no, yes), renal disease (no, yes), chronic pulmonary disease (no, yes), malignant cancer (no, yes), Acute Physiology and Chronic Health Evaluation (APACHE) III score, Sequential Organ Failure Assessment (SOFA) score, GCS score; invasive mechanical ventilation (invasive vent), metformin use; temperature, heart rate, respiratory rate, systolic blood pressure (SBP), diastolic blood pressure (DBP), mean arterial pressure (MAP), creatinine, prothrombin time (PT), partial thromboplastin time (PTT), international normalized ratio (INR), glycated hemoglobin (HbA1c), triglycerides, total cholesterol, HDL-C, LDL-C, mean glucose, time-weighted average blood glucose (TWA-BG), glucose variability (GV glucose), 72-hour blood glucose change (gly_Δ72), glucose-heart rate ratio (SHR), hemoglobin A1c gap (HGI), triglyceride-glucose index (TyG).

In this study, the dynamic glucose variation indices included mean glucose, TWA-BG, GV glucose, and Gly_Δ72, which were derived from blood glucose data collected in ICU with at least three measurements.

Mean glucose: Average blood glucose level during the ICU stay (13).

TWA-BG: For patients with ≥ 3 blood glucose measurements during the ICU stay, the i-th blood glucose measurement was recorded as Gi, and the time interval between the i-th and (i+1)-th blood glucose measurements was recorded as Ti. The last Ti was defined as the time interval between the last Gi and the time of discharge or death (14).


TWAG=∑(Gi×ΔTi)÷∑ΔTi

GV glucose: Ratio of the standard deviation of blood glucose to the mean of all repeated blood glucose measurements during the ICU stay, with each patient having ≥ 3 blood glucose measurements during the ICU stay (15).

Gly_Δ72: Individualized linear regression was performed for each patient’s blood glucose values (Y-axis) and time (X-axis) within 72 hours to calculate the regression slope (β_1_), which was then multiplied by 72 hours to obtain the total blood glucose change (unit: mg/dL/72h). The slope represents the average hourly blood glucose change trend: a positive value indicates an increase in blood glucose, while a negative value indicates a decrease. To avoid the interference of extreme values, the Tukey method was used for truncation: if a patient’s gly_Δ72 exceeded 3 times the interquartile range (IQR) (i.e., < Q1 - 3×IQR or > Q3 + 3×IQR), it was replaced with the corresponding lower or upper limit.

SHR: Calculated as follows: SHR = immediate blood glucose at admission (mmol/L)/[(1.59 × HbA1c (%)) - 2.59] (16).

HGI: Difference between the observed and predicted HbA1c values, where the predicted HbA1c was derived from a linear regression model based on baseline fasting plasma glucose The prediction equation was: Predicted HbA1c = 0.009 × FPG + 4.940 (17).

TyG: Calculated as follows: TyG = ln [fasting triglycerides (mg/dL) × fasting blood glucose (mg/dL)]/2 (18).

### 2.4 Data cleaning and missing data imputation

To reduce bias caused by missing data, variables with > 20% missing values were excluded from this study; data with ≤ 20% missing values were subjected to multiple imputation with five imputation iterations to ensure data integrity for subsequent analysis.

### 2.5 Model construction methods

Based on the cleaned dataset, ML algorithms were applied to construct a predictive model for in-hospital mortality in NTICH patients. The specific steps were as follows:

Dataset splitting

The MIMIC-IV dataset was randomly divided into a training set and an internal validation set at a ratio of 7:3. The training set was used for model construction and parameter optimization, while the validation set was adopted to evaluate the generalization ability of the models.

Feature selection

To minimize the negative impact of overfitting, the Boruta algorithm was used to evaluate the importance of features and remove irrelevant or redundant invalid features.

Model selection and training

ML algorithms including Logistic Regression (LR), eXtreme Gradient Boosting (XGBoost), Light Gradient Boosting Machine (LightGBM), Ridge Regression (RR), Decision Tree (DT), K-Nearest Neighbors (KNN), Random Forest (RF), Multi-Layer Perceptron (MLP), and Support Vector Machine (SVM) were used to construct predictive models for in-hospital mortality in NTICH patients. 5-fold cross-validation was performed on the training set to preliminarily assess model performance.

Model evaluation and validation

The area under the curve (AUC) and decision curve analysis (DCA) were used to evaluate the reliability and clinical application value of the 9 ML models. After a comprehensive comparison of different ML models, the model with the best predictive performance was selected as the final predictive model. To further confirm the applicability of the selected model, it was evaluated in both internal and external validation cohorts.

Model interpretability analysis

Finally, SHapley Additive exPlanations (SHAP) were used to rank the importance of each feature, which enhanced the clinical interpretability of the model.

### 2.6 Statistical analysis

Data cleaning and analysis were performed using R software (Version 4.3.2). Normally distributed quantitative data were expressed as mean ± standard deviation, and intergroup comparisons were performed using analysis of variance. Non-normally distributed quantitative data were expressed as median (interquartile range), and intergroup comparisons were conducted using the Kruskal-Wallis test. Qualitative data were presented as counts (percentages), and intergroup comparisons were made using the chi-square test or Fisher’s exact test. A difference was considered statistically significant when *P* < 0.05.

## 3 Results

### 3.1 Patient characteristics

**Figure 1:**
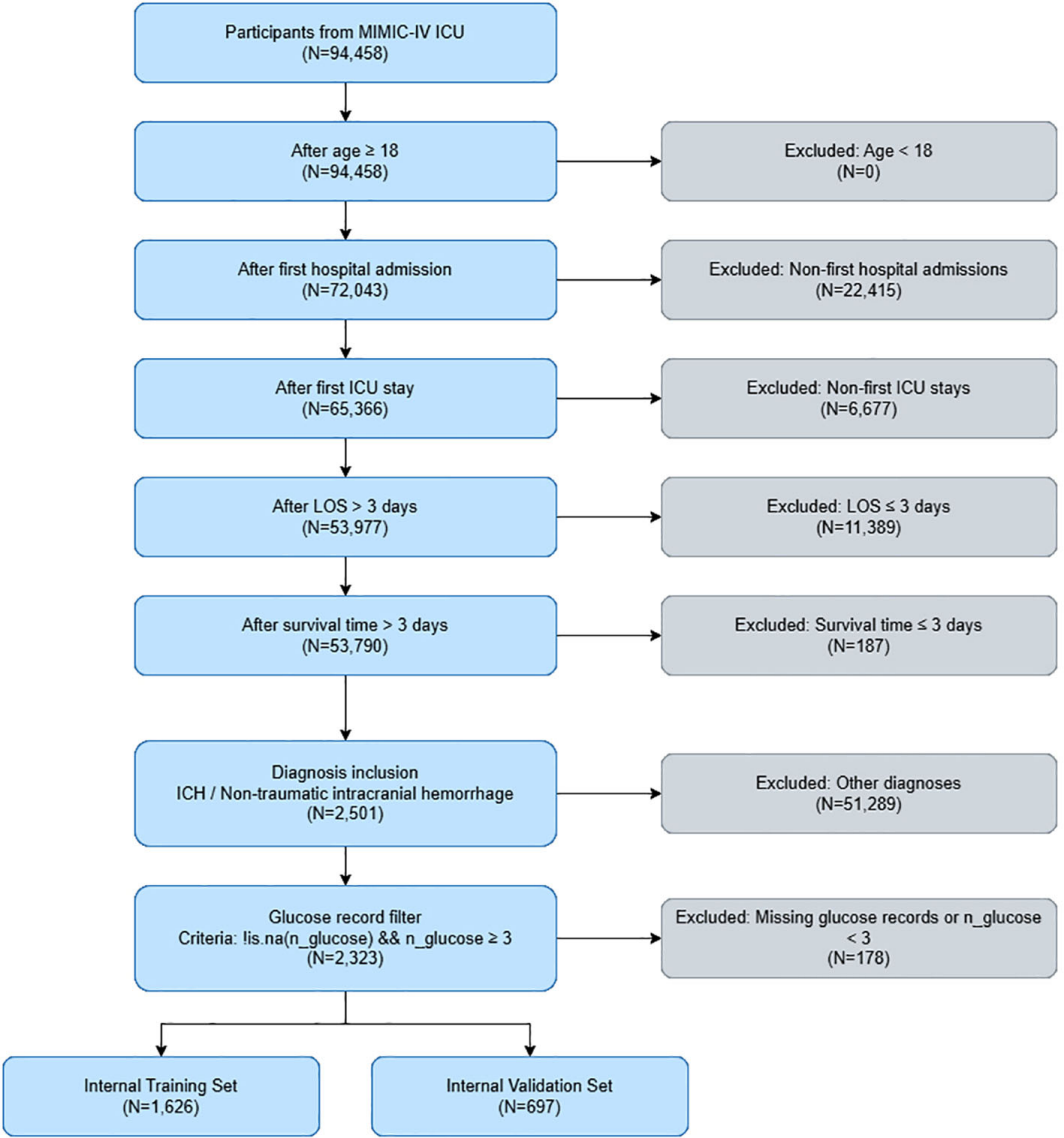
Participant screening flow diagram.

A total of 2,323 NTICH patients were included from the MIMIC-IV database (**Figure 1**), among whom 326 patients died in-hospital (14.03%). Compared with the non-in-hospital mortality group, the in-hospital mortality group was older (70.9 ± 14.36 vs. 67.9 ± 15.54, *P* = 0.001) and had significantly higher proportions of diabetes (34.0% vs. 25.3%, *P* = 0.001), myocardial infarction (12.6% vs. 8.2%, *P* = 0.013), congestive heart failure (19.9% vs. 12.9%, *P* = 0.001), and renal disease (19.0% vs. 11.2%, *P* < 0.001). For lipid parameters, total cholesterol was significantly lower in the in-hospital mortality group (152.88 ± 42.13 vs. 165.23 ± 43.89, *P* < 0.001), along with lower HDL-C (48.07 ± 16.71 vs. 51.17 ± 17.33, *P* = 0.003) and LDL-C (88.64 ± 38.72 vs. 94.46 ± 38.36, *P* = 0.011), whereas triglycerides were higher in the in-hospital group (159.09 ± 140.60 vs. 128.89 ± 111.95, *P* < 0.001). Regarding glucose indicators, the SHR was significantly higher in the in-hospital mortality group (1.22 ± 0.38 vs. 1.10 ± 0.30, *P* < 0.001), as was the TyG (9.15 ± 0.71 vs. 8.83 ± 0.67, *P* < 0.001) and HbA1c (6.38 ± 1.52 vs. 6.08 ± 1.29, *P* < 0.001). Significant differences were also observed between the two groups in terms of race (*P* < 0.001), APACHE III score (*P* < 0.001), SOFA score (*P* < 0.001), GCS score (*P* < 0.001), invasive vent (*P* < 0.001), metformin use (*P* = 0.004), heart rate (*P* = 0.019), respiratory rate (*P* < 0.001), DBP (*P* = 0.016), creatinine (*P* < 0.001), PT (*P* < 0.001), PTT (*P* = 0.002), and INR (*P* = 0.004) (**Table 1**).

**Table 1:** Baseline characteristics of patients in the MIMIC-IV database.

Variable	Level	No	Yes	*P*
n		1997	326	
Sex (%)	Female	901 (45.1)	145 (44.5)	0.877
	Male	1096 (54.9)	181 (55.5)	
Age (mean (SD))		67.86 (15.54)	70.92 (14.36)	0.001
Marital status (%)	Divorced	147 (7.4)	21 (6.4)	0.307
	Married	1093 (54.7)	186 (57.1)	
	Single	518 (25.9)	72 (22.1)	
	Widowed	239 (12.0)	47 (14.4)	
Race (%)	Black	189 (9.5)	32 (9.8)	<0.001
	Other	598 (29.9)	142 (43.6)	
	White	1210 (60.6)	152 (46.6)	
Height (mean (SD))		168.27 (10.68)	167.72 (10.95)	0.384
Weight (mean (SD))		79.95 (31.90)	77.80 (21.16)	0.241
Smoker (%)	No	1907 (95.5)	311 (95.4)	1.000
	Yes	90 (4.5)	15 (4.6)	
Alcohol abuse (%)	No	1980 (99.1)	324 (99.4)	0.912
	Yes	17 (0.9)	2 (0.6)	
Hypertension (%)	No	759 (38.0)	139 (42.6)	0.126
	Yes	1238 (62.0)	187 (57.4)	
Diabetes (%)	No	1492 (74.7)	215 (66.0)	0.001
	Yes	505 (25.3)	111 (34.0)	
Myocardial infarction (%)	No	1833 (91.8)	285 (87.4)	0.013
	Yes	164 (8.2)	41 (12.6)	
Congestive heart failure (%)	No	1739 (87.1)	261 (80.1)	0.001
	Yes	258 (12.9)	65 (19.9)	
Renal disease (%)	No	1774 (88.8)	264 (81.0)	<0.001
	Yes	223 (11.2)	62 (19.0)	
Chronic pulmonary disease (%)	No	1742 (87.2)	280 (85.9)	0.562
	Yes	255 (12.8)	46 (14.1)	
Malignant cancer (%)	No	1777 (89.0)	297 (91.1)	0.293
	Yes	220 (11.0)	29 (8.9)	
APACHE III score (mean (SD))		36.24 (14.73)	49.36 (23.21)	<0.001
SOFA score (mean (SD))		4.21 (2.54)	7.48 (3.87)	<0.001
GCS score (mean (SD))		10.95 (3.36)	7.77 (4.26)	<0.001
Invasive vent (%)	No	1269 (63.5)	67 (20.6)	<0.001
	Yes	728 (36.5)	259 (79.4)	
Metformin use (%)	No	1941 (97.2)	326 (100.0)	0.004
	Yes	56 (2.8)	0 (0.0)	
Temperature (mean (SD))		36.84 (0.59)	36.83 (0.78)	0.823
Heart rate (mean (SD))		82.52 (16.86)	84.95 (19.86)	0.019
Respiratory rate (mean (SD))		18.53 (4.98)	19.62 (5.49)	<0.001
SBP (mean (SD))		137.78 (22.34)	136.56 (22.81)	0.361
DBP (mean (SD))		75.98 (17.58)	73.42 (18.74)	0.016
MBP (mean (SD))		92.77 (17.61)	90.80 (17.42)	0.060
Creatinine (mean (SD))		1.05 (1.17)	1.49 (1.60)	<0.001
PT (mean (SD))		13.40 (5.86)	14.81 (9.03)	<0.001
PTT (mean (SD))		30.17 (12.62)	32.67 (17.59)	0.002
INR (mean (SD))		1.22 (0.50)	1.30 (0.46)	0.004
HbA1C (mean (SD))		6.08 (1.29)	6.38 (1.52)	<0.001
Triglycerides (mean (SD))		128.89 (111.95)	159.09 (140.60)	<0.001
Total cholesterol (mean (SD))		165.23 (43.89)	152.88 (42.13)	<0.001
HDL-C (mean (SD))		51.17 (17.33)	48.07 (16.71)	0.003
LDL-C (mean (SD))		94.46 (38.36)	88.64 (38.72)	0.011
TWA-BG (mean (SD))		153.87 (588.50)	156.81 (38.61)	0.928
GV glucose (mean (SD))		0.20 (0.30)	0.23 (0.21)	0.081
Gly_delta72 (mean (SD))		-3.01 (88.23)	1.21 (91.53)	0.426
Mean glucose (mean (SD))		157.05 (612.62)	156.38 (37.33)	0.984
SHR (mean (SD))		1.10 (0.30)	1.22 (0.38)	<0.001
HGI (mean (SD))		-0.07 (1.11)	0.02 (1.31)	0.209
TyG (mean (SD))		8.83 (0.67)	9.15 (0.71)	<0.001

MIMIC, Medical Information Mart for Intensive Care; APACHE III, Acute Physiology and Chronic Health Evaluation III; SOFA, Sequential Organ Failure Assessment; GCS, Glasgow Coma Scale; invasive vent, invasive ventilation; SBP, systolic blood pressure; DBP, diastolic blood pressure; MBP, mean arterial pressure; PT, prothrombin time; PTT, partial thromboplastin time; INR, international normalized ratio; HbA1C, glycated hemoglobin A1C; HDL-C, high-density lipoprotein cholesterol; LDL-C, low-density lipoprotein cholesterol; TWA-BG, time-weighted average blood glucose; GV glucose, glucose variability; Gly_delta72, 72-hour blood glucose change; SHR, glucose-heart rate ratio; HGI, hemoglobin A1C gap; TyG, triglyceride-glucose index.

### 3.2 Feature selection

**Figure 2:**
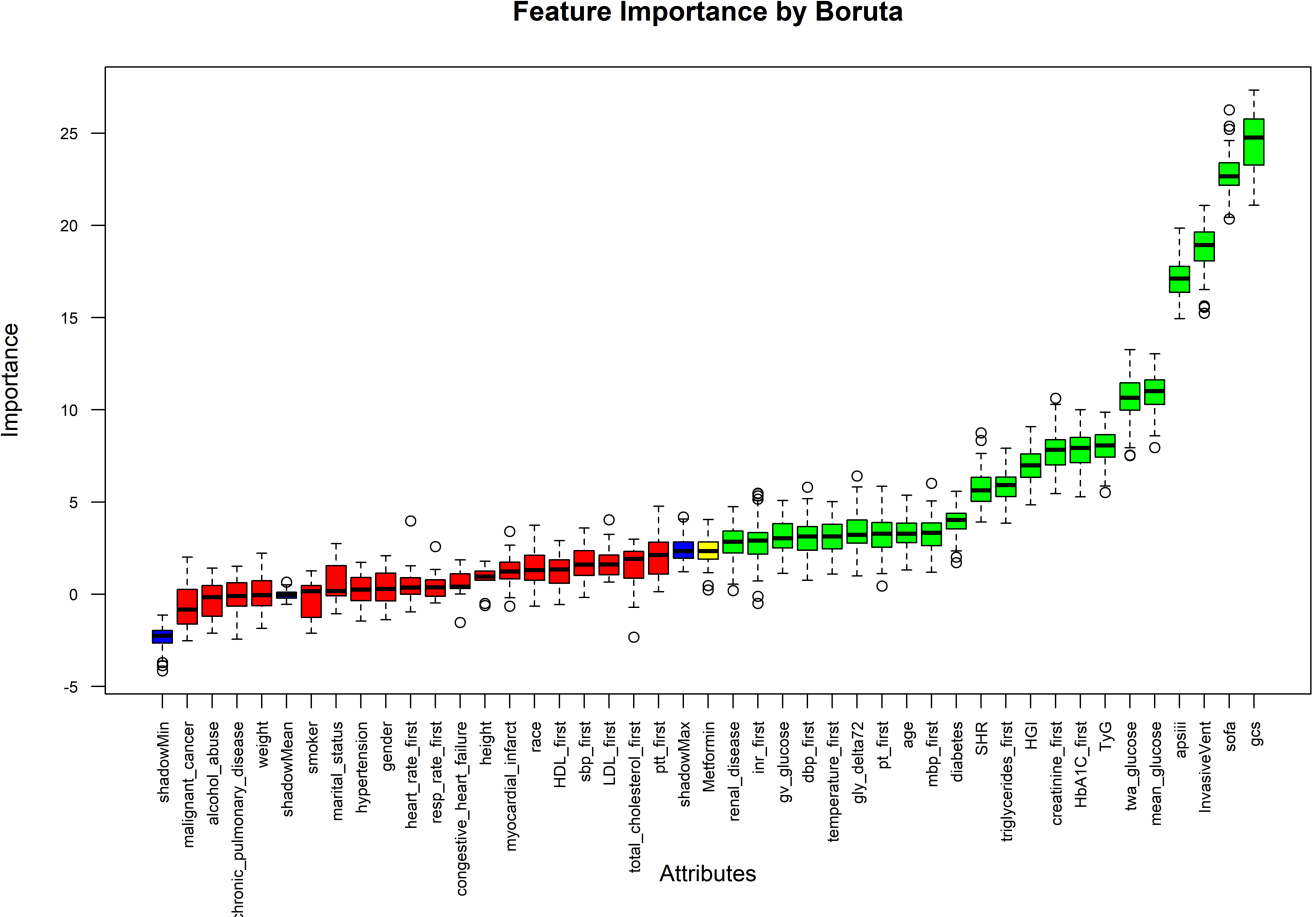
Feature selection by Boruta.

The Boruta algorithm was used to initially screen the predictive factors for NTICH patients, which included GCS score, SOFA score, invasive vent, APACHE III score, mean glucose, TWA-BG, TyG, HbA1c, creatinine, HGI, triglycerides, SHR, diabetes, MBP, age, PT, gly_Δ72, temperature, DBP, GV glucose, INR, and renal disease (**Figure 2**).

### 3.3 Predictive models

**Figure 3:**
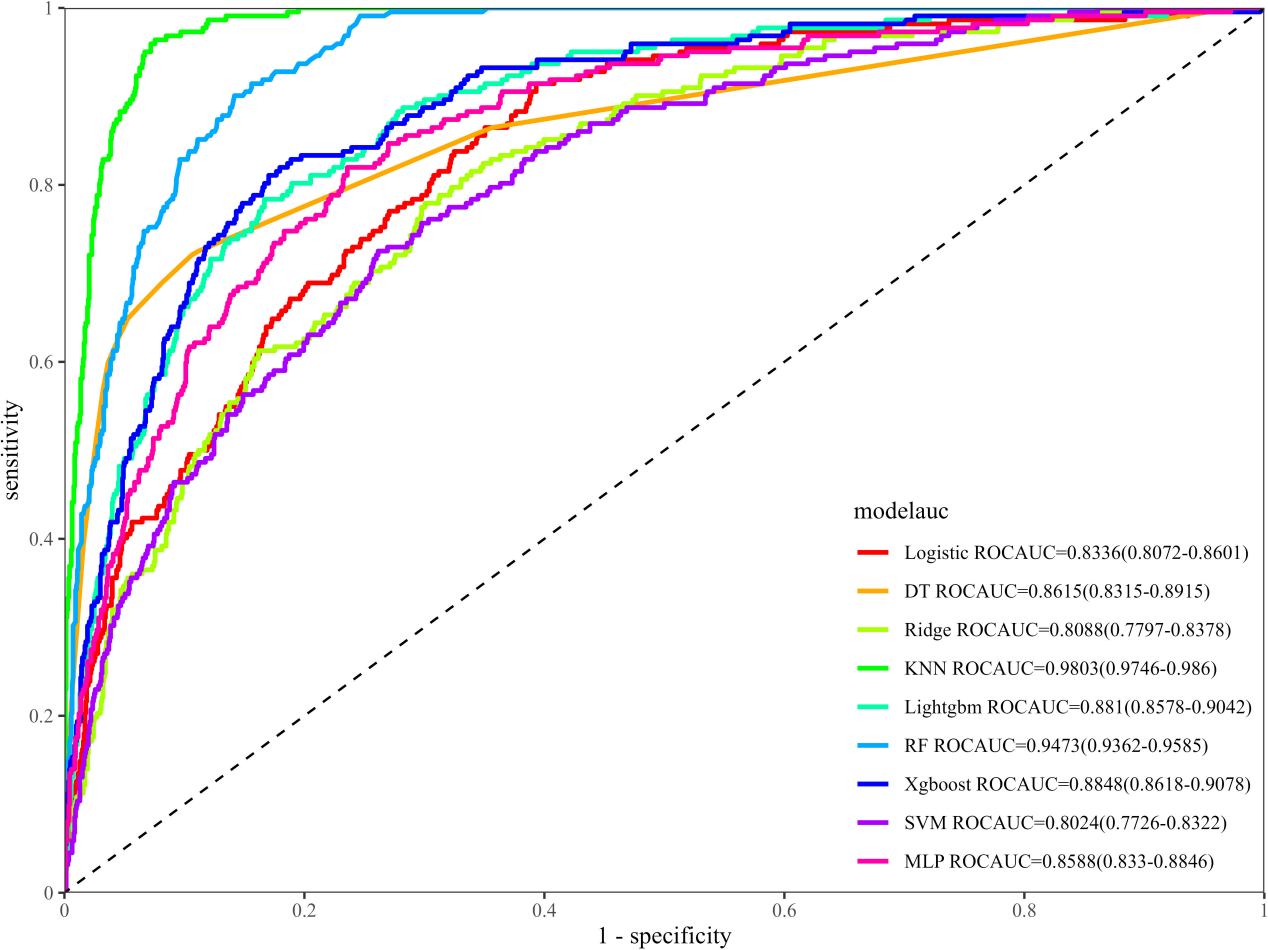
ROC curve of the training set. ROC, Receiver Operating Characteristic.

**Figure 4:**
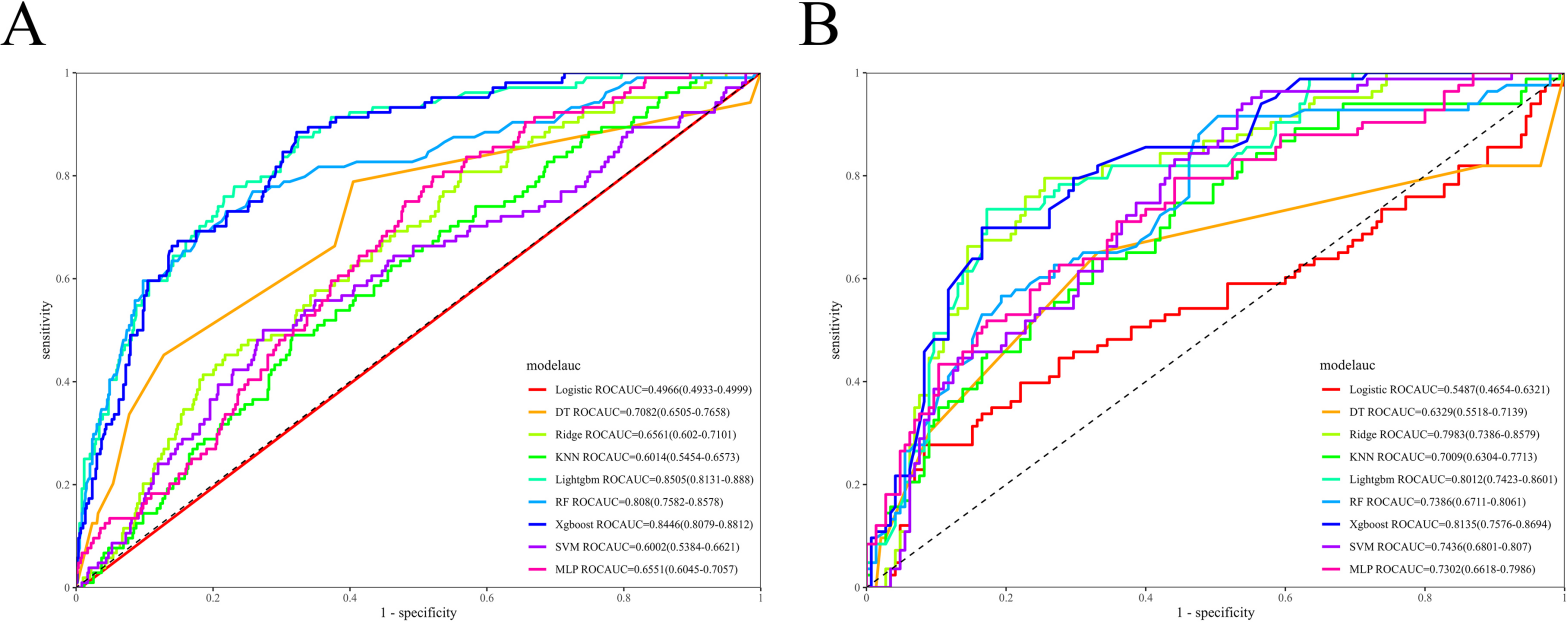
ROC curves of the validation set. **(A)** Internal validation set; **(B)** External validation set. ROC, Receiver Operating Characteristic; LR, Logistic Regression; XGBoost, eXtreme Gradient Boosting; LightGBM, Light Gradient Boosting Machine; RR, Ridge Regression; DT, Decision Tree; KNN, K-Nearest Neighbors; RF, Random Forest; MLP, Multi-Layer Perceptron; and SVM, Support Vector Machine.

In the training set, the KNN model showed the best performance, with an AUC of 0.980 (95% CI: 0.975–0.986); followed by the RF model, XGBoost model, and LightGBM model, with the AUCs of 0.947 (95% CI: 0.936–0.959), 0.885 (95% CI: 0.862–0.908), and 0.881 (95% CI: 0.858–0.904), respectively (**Figure 3**). In the internal validation set, the LightGBM model performed best, with an AUC of 0.851 (95% CI: 0.813–0.888), followed by XGBoost (AUC = 0.845, 95% CI: 0.808–0.881) and RF (AUC = 0.808, 95% CI: 0.758–0.858) (**Figure 4A**). In the external validation set, the XGBoost model achieved the highest AUC of 0.814 (95% CI: 0.758–0.869), followed by LightGBM (AUC = 0.801, 95% CI: 0.742–0.860) and RR (AUC = 0.798, 95% CI: 0.739–0.858) (**Figure 4B**).

**Figure 5:**
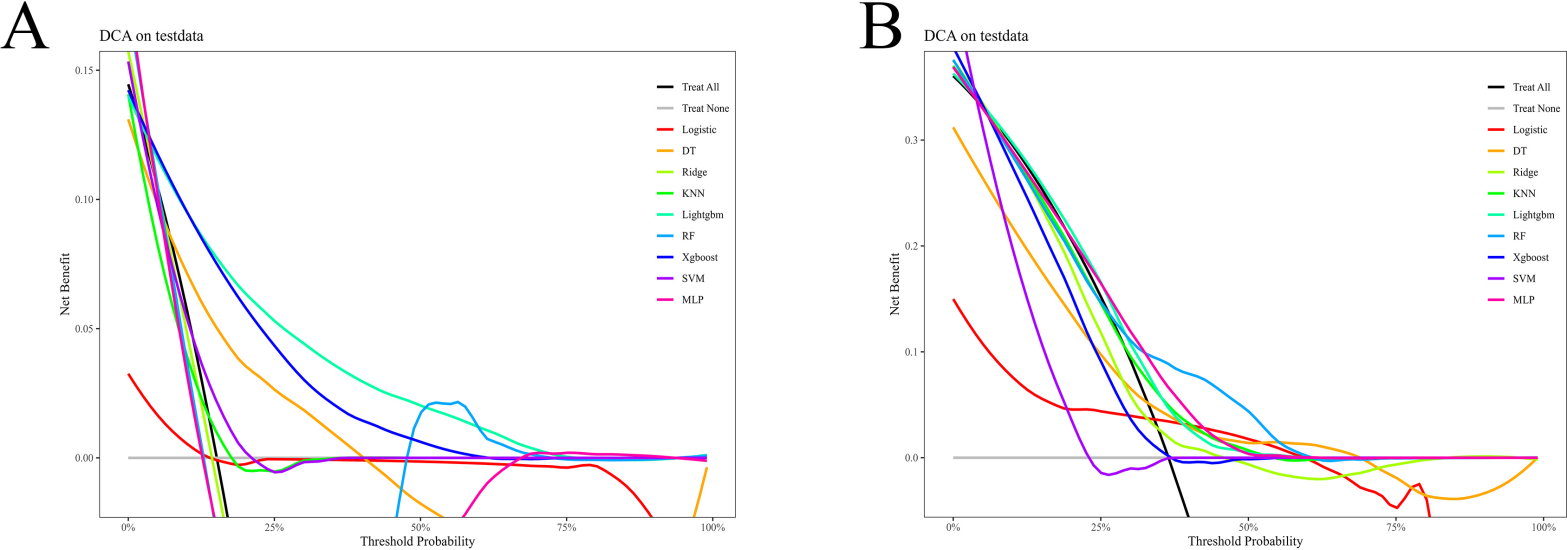
DCA curves of the validation set. **(A)** Internal validation set; **(B)** External validation set. DCA, decision curve analysis; LR, Logistic Regression; RR, Ridge Regression; DT, Decision Tree; KNN, K-Nearest Neighbors; RF, Random Forest; MLP, Multi-Layer Perceptron; and SVM, Support Vector Machine.

In the internal validation set, within the threshold range of 0%–25%, the clinical net benefit of models such as KNN and MLP was significantly higher than that of the “Treat All” and “Treat None” strategies; within the threshold range of 0%–75%, the clinical net benefit of models such as LightGBM, and XGBoost was significantly higher than that of “Treat All” and “Treat None” strategies (**Figure 5A**). In the external validation set, within the threshold range of 30%–60%, the clinical net benefit of models such as RF, MLP, LightGBM, and KNN was significantly higher than that of “Treat All” and “Treat None” strategies (**Figure 5B**).

### 3.4 Interpretation of ML models

**Figure 6:**
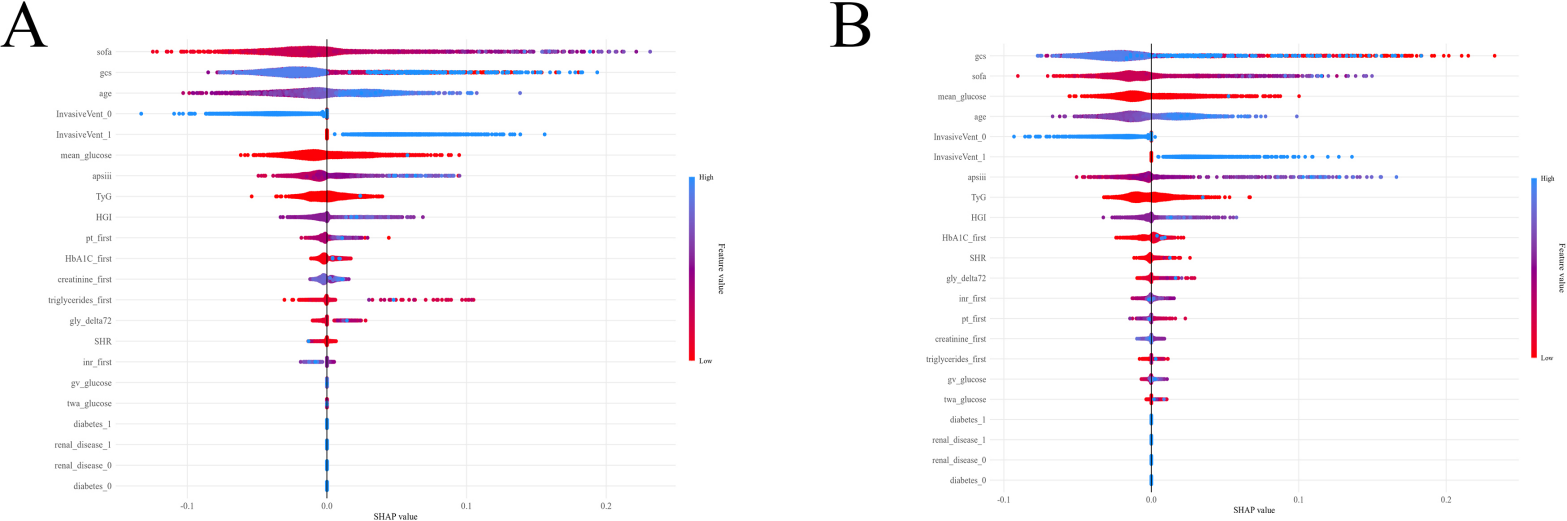
SHAP Plots of LightGBM and XGBoost. **(A)** LightGBM; **(B)** XGBoost SHAP, SHapley Additive exPlanations; LightGBM, Light Gradient Boosting Machine; XGBoost, eXtreme Gradient Boosting.

SHAP plots revealed how each feature influenced the model’s predictions. In the LightGBM model, the SOFA score topped the key features influencing the prediction results, succeeded by the GCS score, age, invasive vent, and mean glucose (**Figure 6A**). In the XGBoost model, the GCS score ranked first among the key features driving the predictions, followed by the SOFA score, mean glucose, age, and invasive vent. (**Figure 6B**).

## 4 Discussion

Based on data from the MIMIC-IV database and our institution, this study integrated dynamic glucose variation indices, lipid profiles, and clinical characteristics to construct a ML-based predictive model for in-hospital mortality in NTICH patients. It revealed the association between dynamic glucose variation indices, lipid profiles, and prognosis, and verified the clinical utility of the model. The results not only provided new biomarkers and technical tools for NTICH prognostic assessment but also offered support for clinical individualized management.

In the feature selection phase, this study adopted the Boruta algorithm, which has unique advantages in the construction of clinical predictive models. Boruta is a wrapper-based all-relevant feature selection algorithm built on random forests. Its core advantage lies in creating random “shadow features” as statistical reference benchmarks, which enables the systematic identification of all features truly correlated with the outcome variable while effectively eliminating noise and redundant variables from high-dimensional data (19). Compared with traditional filter or embedded methods, the “all-relevant” principle of Boruta is particularly suitable for exploratory clinical research. Its goal is not merely to construct a parsimonious predictive model, but to unbiasedly identify all potential predictive factors, a feature that is crucial for mining novel biomarkers from large-scale clinical datasets (20). Recent studies have confirmed that the Boruta algorithm significantly improves the reliability of clinical predictive models and the potential for biomedical discoveries by providing stable and interpretable feature rankings, thus laying a methodological foundation for integrating multi-dimensional data to build high-performance models (21, 22).

This study compared the predictive performance of multiple ML algorithms and found that the KNN model performed best in the training set (AUC = 0.980), while the XGBoost and LightGBM models exhibited more stable generalization ability in the internal and external validation sets (AUCs of 0.814 and 0.801 in the external validation set, respectively). These results reflect the characteristics of different algorithms: KNN, as a lazy learning algorithm, demonstrates high fitting accuracy in the training set but poor adaptability to unseen data; in contrast, XGBoost and LightGBM effectively mitigate the risk of overfitting through ensemble learning strategies and are better suitable for handling high-dimensional and non-linear relationships in clinical data (23, 24). In a study predicting in-hospital mortality in heart failure patients, XGBoost outperformed Classification and Regression Trees (CART) and Random Forest (RF), with an AUC of 0.816 (95% CI: 0.815–0.818) (25). Another study based on the MIMIC-IV database demonstrated that among 8 ML models, XGBoost achieved the highest predictive performance for in-hospital mortality in patients with acute kidney injury after ICU admission, with an AUC of 0.833 (26). Compared with traditional prognostic tools (GCS Score), the model constructed in this study offers three key advantages: first, in terms of multi-dimensional feature integration, it not only incorporates traditional clinical indicators such as GCS and SOFA scores but also innovatively integrates dynamic glucose variation indices (e.g., mean glucose, TWA-BG, GV glucose, gly_Δ72) and comprehensive lipid profiles (HDL-C, triglycerides). This integration more comprehensively captures the characteristics of metabolic disorders during NTICH progression, as NTICH-induced brain injury disrupts the hypothalamic-pituitary-adrenal axis and the sympathetic nervous system, leading to secondary hyperglycemia and lipid metabolism disorders, which in turn further exacerbates cerebral edema and blood-brain barrier disruption (27). Second, regarding the combination of dynamic and long-term metabolic assessment, traditional models rely solely on static indicators at admission, while indicators such as TyG and HbA1c incorporated in this study can reflect medium- and long-term metabolic status. This is particularly critical for NTICH, as pre-existing metabolic abnormalities (e.g., insulin resistance) can impair cerebral vascular autoregulation and increase the vulnerability of brain tissue to ischemic damage after hemorrhage, while acute blood glucose fluctuations during hospitalization further amplify neuroinflammation (28). Thus, integrating both short-term dynamic and long-term metabolic indicators aligns with the dynamic evolution of metabolic disorders throughout the NTICH course (28). Third, in terms of clinical practicality, DCA confirmed that within different threshold probability ranges, the net benefit of models such as XGBoost and LightGBM was significantly higher than that of the “Treat All” or “Treat None” strategies, suggesting that these models can assist clinicians in making more precise intervention decisions under different risk thresholds. Specifically, at a threshold probability of 30%–60%, these models yielded greater net benefits, indicating their ability to assist clinicians in stratifying patients into distinct risk groups: high-risk patients may require intensified monitoring and early intervention, while low-risk patients can avoid unnecessary overtreatment, directly addresses the unmet need for precision risk stratification in the clinical management of NTICH. Nevertheless, it is important to note that the model performance in this study may be limited by the quality of the data itself and the depth of feature engineering. Although this study innovatively integrated dynamic glucose variation indices and lipid profiles, more complex temporal feature extraction methods could be explored in future research to more precisely capture the dynamic evolution process of metabolic indicators and improve prediction accuracy.

By quantifying the contribution of each feature to the model’s predictions, SHAP plots identified the core factors affecting the in-hospital mortality risk of NTICH patients, providing key evidence for the interpretability and clinical application of the model. This addresses a critical requirement for translating ML models into clinical practice, as “black box” models are often met with skepticism by clinicians. In this study, the SHAP analysis results of the LightGBM and XGBoost models were highly consistent, both indicating that SOFA score, GCS score, age, invasive vent, and mean glucose were the main features driving prognostic predictions, with the direction of their effects closely aligning with clinical pathophysiological mechanisms. The SOFA score and GCS score are core indicators reflecting disease severity. SHAP values showed that SOFA score and GCS score had the highest weights in both models, suggesting that organ dysfunction and consciousness status are the most direct predictive factors for in-hospital mortality in NTICH (29). Specifically, an increase in SOFA score and a decrease in GCS score contributed the most to the prediction of in-hospital death, which is highly consistent with previous research findings. A study using ML models to assess mortality in NTICH found that the “GCS Motor” feature ranked first in importance among multiple features (12). In another ML-based study, the most important feature affecting in-hospital mortality in patients with spontaneous ICH in the ICU was the GCS score, followed by the SOFA score (29). Mechanistically, NTICH can trigger a systemic inflammatory response syndrome (SIRS) through the release of pro-inflammatory cytokines (TNF-α, IL-6) from damaged brain tissue, which disrupts the function of distant organs (e.g., lungs, kidneys, heart) and forms a vicious cycle of “brain-organ crosstalk” vicious cycle (30). An elevated SOFA score directly reflects the extent of this multi-organ dysfunction, which further impairs cerebral perfusion and accumulates metabolic waste, exacerbating primary brain injury. Conversely, a decreased GCS score indicates severe damage to the cerebral cortex or brainstem, which not only reflects the severity of primary hemorrhage but also increases the risk of secondary complications (aspiration pneumonia, deep vein thrombosis, hematoma expansion), all of which amplify the inflammatory response and worsen prognosis (31, 32). Age and invasive vent reflect physiological reserve and disease severity. As an independent predictive factor, age showed a stable positive contribution in the SHAP plots. The elderly population is inherently more vulnerable to NTICH due to age-related cerebrovascular changes (arteriosclerosis, reduced vascular elasticity), declined organ function (impaired renal clearance, decreased cardiac output), and compromised stress response capacity (33). Additionally, elderly NTICH patients often have comorbidities (diabetes mellitus, hypertension, coronary artery disease) that further reduce their tolerance to hemorrhage and secondary organ damage, leading to a higher risk of mortality (34, 35). The use of invasive vent was also a strong predictive factor, and its SHAP value indicates a significant positive correlation with in-hospital mortality risk. Invasive ventilation is a rescue measure for NTICH patients with respiratory failure or severe consciousness disturbance, and its application itself signifies critical illness (36). Moreover, invasive ventilation can induce ventilator-associated pneumonia or ventilator-induced lung injury, which further exacerbates systemic inflammation and multi-organ dysfunction, creating a secondary injury cascade that increases mortality risk (37). Mean glucose reflects the overall short-term and continuous blood glucose exposure, enabling more accurate capture of the cumulative effect of daily blood glucose fluctuations. Short-term hyperglycemia can rapidly induce stress injury in cerebrovascular endothelial cells, leading to a sharp increase in reactive oxygen species, a decrease in vasodilators, and vascular wall stiffness; simultaneously, it impairs the blood-brain barrier, degrades tight junction proteins, and increases its permeability. Additionally, it activates microglia to release pro-inflammatory factors, exacerbates cerebrovascular vulnerability, and ultimately increases in-hospital mortality (38, 39).

The clinical significance of dynamic glucose variation indices and lipid profiles in patients with NTICH lies in that they provide key metabolic insights from two distinct dimensions: acute fluctuations and chronic baseline status. Dynamic glucose variation indices (e.g., mean glucose, TWA-BG, GV glucose, Gly_Δ72) can dynamically capture blood glucose fluctuations and cumulative exposure levels during hospitalization. Stress-induced hyperglycemia and its drastic fluctuations following acute brain injury are more predictive of poor prognosis than a single admission blood glucose measurement. Studies have demonstrated that blood glucose fluctuations can exacerbate cerebral edema and secondary brain injury by enhancing oxidative stress, disrupting blood-brain barrier integrity, and amplifying neuroinflammatory responses, thereby increasing mortality risk (40). Santana et al. systematically reviewed the challenges of glycemic management during early brain injury after aneurysmal subarachnoid hemorrhage and prospectively highlighted the potential value of adopting dynamic glucose variation indices (7). Our clinical findings indirectly validate this perspective, providing preliminary evidence for the feasibility and necessity of its clinical application. Lipid profiles, by contrast, reflect patients’ baseline metabolic status. Recent clinical studies have confirmed that low levels of triglycerides, total cholesterol, and LDL-C, as well as HDL-C, are independently associated with poor functional outcomes after spontaneous intracerebral hemorrhage (41). This may be ascribed to the fact that dyslipidemia impairs cerebrovascular integrity, modulates inflammatory responses, and elevates the risk of hematoma expansion. Integrating these two categories of indicators enables a more comprehensive assessment of the full spectrum of metabolic disorders in NTICH patients, furnishing a richer set of biomarkers for prognostic evaluation. Tang et al. verified in a multicenter cohort study that the TyG index, an indicator integrating lipid and glucose metabolic disorders, was positively correlated with intracerebral hemorrhage and serves as an effective and stable marker for clinical monitoring (42). To improve patient prognosis, future clinical practice should prioritize the integration of dynamic glucose variation indices (e.g., mean glucose, TWA-BG, GV glucose, Gly_Δ72) into the routine management of critically ill NTICH patients, so as to achieve real-time and refined glycemic control. The core goal is not merely to alleviate hyperglycemia, but also to minimize blood glucose variability. On this basis, early and individualized metabolic interventions should be implemented, for instance, initiating intensive management protocols for high-risk patients based on risk assessments that incorporate the TyG index, HbA1c, and CGM metrics. Meanwhile, it is worth exploring the safety of targeted interventions (e.g., statin therapy) for patients with specific lipid metabolic disorders during the post-acute phase, as well as their impacts on neurological function recovery. Ultimately, we aim to facilitate the translation of multimodal predictive models incorporating these metabolic indicators into clinical decision-support tools. Through precise risk stratification, this approach will enable the early implementation of interventions and optimized allocation of medical resources, thereby improving patient outcomes.

This study has several limitations that should be acknowledged. First, it employed a retrospective design, which is subject to selection bias. Moreover, it cannot establish a causal relationship between variables and in-hospital mortality, but can only reflect an association. Future prospective studies are needed to further verify the findings. In addition, the MIMIC-IV database is derived from a single center, and external validation was only based on cases from one hospital, resulting in narrow geographical and population coverage. This may lead to a decline in model performance when extrapolated to other medical centers or populations. Future studies should expand the geographical coverage and include multicenter (including hospitals of different levels) and multi-ethnic prospective cohorts to further improve the generalizability of the model.

## 5 Conclusion

The ML models based on XGBoost/LightGBM showed excellent performance in predicting in-hospital mortality in NTICH patients. SOFA score, GCS score, age, invasive ventilation, and mean glucose were the main factors affecting in-hospital mortality in NTICH patients. This study facilitates the accurate identification of high-risk patients and stratified allocation of medical resources in neurocritical care clinical practice.

## Data Availability

The original contributions presented in the study are included in the article/supplementary material, further inquiries can be directed to the corresponding authors.
